# Co-Expression of a Homologous Cytochrome P450 Reductase Is Required for In Vivo Validation of the *Tetranychus urticae* CYP392A16-Based Abamectin Resistance in *Drosophila*

**DOI:** 10.3390/insects11120829

**Published:** 2020-11-25

**Authors:** Maria Riga, Aris Ilias, John Vontas, Vassilis Douris

**Affiliations:** 1 Institute of Molecular Biology & Biotechnology, Foundation for Research & Technology Hellas, 100 N. Plastira Street, GR-700 13 Heraklion, Greece; maria_riga@imbb.forth.gr (M.R.); aris_ilias@imbb.forth.gr (A.I.); 2 Laboratory of Pesticide Science, Department of Crop Science, Agricultural University of Athens, 75 Iera Odos Street, GR-118 55 Athens, Greece; 3 Department of Biological Applications and Technology, University of Ioannina, GR-45 110 Ioannina, Greece

**Keywords:** detoxification, cytochrome P450, cytochrome P450 reductase, abamectin, *Tetranychus*, transgenic *Drosophila*

## Abstract

**Simple Summary:**

The two-spotted spider mite, *Tetranychus urticae*, is one of the most damaging agricultural pests worldwide, feeding on over 1100 plant species and causing extensive damage to several crops. Chemical acaricides remain the most widely used strategy to control this pest. However, *T. urticae* has developed significant resistance to numerous acaricide compounds, due to certain features of mite biology and extensive acaricide applications that lead to the selection of resistant pests and subsequently the emergence of resistant populations. Several molecular/genetic mechanisms may contribute to these highly resistant phenotypes. Such mechanisms frequently involve expression of P450 detoxification enzymes, which act together with a partner protein named cytochrome P450 reductase (CPR). In this study, we investigated the potential of a mite P450 enzyme, CYP392A16, to confer resistance to the acaricide abamectin in vivo, when expressed in tissues of the model fruit fly *Drosophila melanogaster*. We confirmed that expression of this enzyme contributes to abamectin resistance in the fruit fly model, but only when a homologous mite CPR is co-expressed. Our findings indicate that the *Drosophila* model system can be engineered to facilitate validation of the candidate mite P450s, in order to elucidate resistance mechanisms and their underlying interactions.

**Abstract:**

Overexpression of the cytochrome P450 monooxygenase CYP392A16 has been previously associated with abamectin resistance using transcriptional analysis in the two-spotted spider mite *Tetranychus urticae*, an important pest species worldwide; however, this association has not been functionally validated in vivo despite the demonstrated ability of CYP392A16 to metabolize abamectin in vitro. We expressed CYP392A16 in vivo via a Gal4 transcription activator protein/Upstream Activating Sequence (GAL4/UAS) system in *Drosophila melanogaster* flies, driving expression with detoxification tissue-specific drivers. We demonstrated that CYP392A16 expression confers statistically significant abamectin resistance in toxicity bioassays in *Drosophila* only when its homologous redox partner, cytochrome P450 reductase (TuCPR), is co-expressed in transgenic flies. Our study shows that the *Drosophila* model can be further improved, to facilitate the functional analysis of insecticide resistance mechanisms acting alone or in combination.

## 1. Introduction

The two-spotted spider mite, *Tetranychus urticae* (Koch), is one of the most damaging agricultural pests worldwide, utilizing over 1100 plant species as hosts [1,2]. For several years, its control has been based mostly on the use of chemical acaricides [3]. However, *T. urticae* has developed high levels of resistance to numerous acaricide compounds, due both to its biology (arrhenotokous reproduction, short life-cycle, and high fecundity) and to strong acaricide selection pressure [4,5]. Acaricide resistance in *T. urticae* has been associated with different mechanisms, including target-site mutations [6,7,8,9,10,11,12,13,14,15,16,17,18,19] and enhanced detoxification [20] through the overexpression of different classes of metabolic proteins, such as cytochrome P450s [17,19,21,22,23,24], glutathione S-transferases [25,26], carboxylesterases [27], ABC transporters [28], and UDP-glycosyltransferases [17,29].

One of the most commonly used acaricides in recent years is abamectin, a compound that belongs to the avermectin subfamily of macrocyclic lactones [30]. Avermectins were registered and widely used for several decades as antiparasitic drugs for animal health applications. Abamectin was later developed as an acaricide/insecticide, since it has a broad spectrum of activity against arthropods, including major pests from several insect orders, and some mite species including *T. urticae* [30]. The mode of action of abamectin is the activation of glutamate-gated chloride channels (GluCl), a type of channel specific to invertebrates [31]. Target-site resistance to abamectin in *T. urticae* has been attributed to point mutations in different members of the cys-loop ligand-gated chloride channel family [9,11,17]. However, mode of inheritance analysis [11] and marker-assisted backcrossing [14] revealed a polygenic nature of resistance, and subsequent detailed genetic analyses indicate that additional mechanisms operating in highly resistant strains might contribute to the phenotype [14,32]; these mechanisms most notably implicate overexpression of cytochrome P450s [33].

Cytochrome P450s contribute to metabolic resistance in several pests [34]. Eighty-six cytochrome P450 (CYP) genes were detected in the *T. urticae* genome [35], at least three of which were shown to be associated with the abamectin-resistant phenotype of the exceptionally resistant Mar-ab strain [11,20]. CYP function depends on NADPH-dependent cytochrome P450 reductase (CPR) as a co-factor that provides electrons from NADPH to the heme center of P450s [34]. In contrast to the huge variation of the cytochrome P450 gene family, only one CPR gene is identified in each arthropod species. In *T. urticae*, the contribution of TuCPR to acaricide resistance has only recently been investigated; recent bulk segregant analysis and genomic mapping studies in acaricide-resistant *T. urticae* strains [36,37] implicated TuCPR within a potential genomic locus associated with resistance to spirodiclofen, pyridaben, and tebufenpyrad, while RNA interference (RNAi) was used to investigate the role of CPR in resistance to abamectin, bifenthrin, and fenpyroximate [38].

In recent years, functional expression of candidate CYP genes is routinely used for the investigation of their catalytic properties and substrate specificities. Moreover, the integration of the molecular genetics toolbox developed in model systems like *Drosophila* into insecticide resistance research (reviewed in [39,40,41]) has facilitated novel approaches that have significantly contributed to the validation of several candidate mutations conferring target-site resistance [42], P450 genes potentially conferring metabolic resistance [43], or their synergistic interactions [44], in the absence of confounding genetic effects.

In previous studies regarding *T. urticae* candidate CYP genes, we have successfully used heterologous expression in bacteria coupled with in vitro functional metabolic assays, along with heterologous expression in *Drosophila* to demonstrate that CYP392A11 can metabolize cyenopyrafen and other acaricides in vitro and confer resistance to fenpyroximate in vivo [23]. Furthermore, we have functionally expressed CYP392A16 in vitro and demonstrated that it metabolizes abamectin to a substantially less toxic compound (the 24-OH or 26-OH isomer of hydroxyl-abamectin) [22].

In this study, using transgenic heterologous expression in *Drosophila*, we demonstrate that *T. urticae* CYP392A16 is able to confer abamectin resistance in vivo; importantly, however, a significant resistance phenotype manifests only in the context of TuCPR co-expression, indicating that a homologous CPR may be required for in vivo functional expression of spider mite CYP genes in *Drosophila*.

## 2. Materials and Methods

### 2.1. Chemicals

An abamectin formulation (Vertimec 18EC, Syngenta, Basel, Switzerland) was used in the feeding bioassays.

### 2.2. Insects

The *Drosophila* strain *yellow white* (*yw*) and the balancer lines *yw*; *Cy*O/*Sco* (for the 2nd chromosome) and *yw*; TM3 *Sb e*/TM6b *Tb e* are part of the IMBB/FORTH fly facility collection and were provided by Christos Delidakis (IMBB/FORTH and University of Crete), while the multiple balancer strains (*w*; *If*/*Cy*O*wg*lacZ; MKRS *Sb e*/TM6 *Tb e* and +; +/T(2,3)*Cy*/*Tb*) were provided by Maria Monastirioti (IMBB/FORTH). The HR-GAL4 driver line is described in [45]. All flies were typically maintained at 25 °C, 60–70% humidity, with a 12/12 h photoperiod and a standard fly diet [46].

### 2.3. Generation of pUAST.CYP392A16

In order to generate transgenic flies for heterologous expression of *T. urticae* gene CYP392A16 that has been previously associated with abamectin resistance [22], a plasmid vector for transgenic expression, pUAST.CYP392A16, was generated. The cDNA sequences encoding CYP392A16 (TeturID: tetur06g04520) and cytochrome P450 reductase (CPR) (TeturID: tetur18g03390) were isolated as previously described [22]. A BglII fragment from pCW_CYP392A16 [22] containing the CYP392A16 ORF was subcloned into the unique BamHI site of the pUAST vector, as previously described [23], to generate pUAST.CYP392A16, and clones with the correct orientation were sequence-verified using the primers pUASTF and pUASTR (Table 1).

Generation of the transgenic UAS.TuCPR fly lines is described in [23]; generation of pUAST.TuCPR by subcloning the TuCPR ORF (TeturID: tetur18g03390) from plasmid pACYC-TuCPR [22] into the pUAST vector is also described in [23].

### 2.4. Construction of the Transgenic Fly Strains

A pUAST.CYP392A16 clone, whose sequence was verified and did not contain any mutations compared to the published genome (TeturID: tetur06g04520), was selected in order to inject preblastoderm embryos of the *D. melanogaster yellow-white* (*yw*) strain using standard transformation techniques. Several independent transformed lines were generated and crossed with balancer stocks for the 2nd (*yw*; *Cy*O/*Sco*) and the 3rd chromosome (*yw*; TM3 *Sb e*/TM6b *Tb e*) and different homozygous lines with insertion of the transgene were established and mapped in the relevant chromosome. Generation of transgenic lines bearing the CPR of *T. urticae* was performed using a similar strategy as described previously [23].

**Figure 1:**
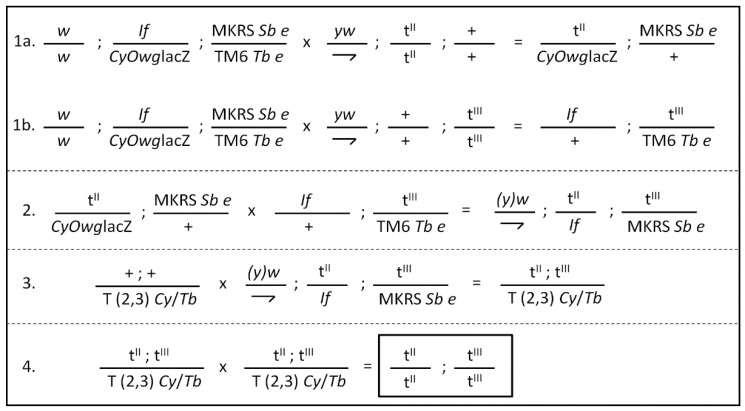
Generic crossing scheme for the generation of strains bearing two transgenes (either HR-GAL4 and UAS-CYP392A16 or UAS-CYP392A16 and UAS-TuCPR) in the 2nd (t^II^) or 3rd (t^III^) chromosome, respectively. Since all types of transgenic flies were originally generated by P-element mediated transgenesis at random (unknown) positions, crosses among several different lines were performed to account for position effects. Virgin multiple-balancer females were crossed with homozygous transgenic males bearing the relevant transgenes at the 2nd chromosome (cross **1a**) or the 3rd chromosome (cross **1b**) and the progeny with the indicated genotype from each cross was used for the cross (**2**) in order to bring both transgenes opposite to the selected markers *If* and *Sb* (note that the chromosomes bearing these markers are not balancers). Male progeny (not undergoing recombination) was crossed with virgin females from a double-balancer strain bearing a rearranged T(2,3) chromosome marked with both *Cy* and *Tb* (cross **3**) and the progeny was selected against *If* and *Sb* to identify individuals expected to have both transgenes opposite to the T(2,3)*Cy*/*Tb* balancer. These were intercrossed (cross **4**) to provide the homozygous strains (shown in box) bearing both UAS-CYP392A16 and UAS-TuCPR or both HR-GAL4 and UAS-CYP392A16, following selection against *Cy*/*Tb* markers. Note that, in the HR-GAL4 line available, the transgene is located at the 2nd chromosome.

In order to generate homozygous transgenic strains that would conditionally express both *CYP392A16* and *TuCPR*, we used lines bearing UAS-CYP392A16 in the 3rd chromosome and lines bearing UAS-TuCPR in the 2nd chromosome and crossed homozygous males with a strain carrying multiple balancer chromosomes (*w*; *If*/*Cy*O*wg*lacZ; MKRS *Sb e*/TM6 *Tb e*) and used standard downstream genetic crosses in order to eventually bring both transgenes against a double-balancer chromosome (Figure 1); these were inter-crossed in order to obtain the line carrying both transgenes in a homozygous state. A similar approach was used to generate lines bearing both HR-GAL4 (at the 2nd chromosome) and UAS-CYP392A16 (at the 3rd chromosome).

### 2.5. Expression of CYP392A16 and/or TuCPR in D. Melanogaster

We employed the GAL4/UAS system to express *CYP392A16* and *TuCPR* in the transgenic flies, as previously described [23,47]. The HR-GAL4 driver [45] was used to drive the expression of *CYP392A16* and/or *TuCPR* in specific tissues relevant to detoxification (Malpighian tubules, midgut, and fat body) [48]. To this end, a series of crosses was performed, as shown in Table 2. Transgenic UAS-CYP392A16 or UAS-TuCPR;UAS-CYP392A16 virgin females were crossed with HR-GAL4 or HR-GAL4;UAS-CYP392A16 males in order to generate progeny that bears the HR-GAL4 transgene, driving expression of CYP392A16 alone or along with TuCPR, as shown in Table 2 (“Transgene dosage” columns). This progeny was used in toxicity bioassays (see below), in comparison with progeny from the cross of *yw* virgin females with HR-GAL4 males (i.e., not driving transgene expression), which served as the negative control.

### 2.6. Extraction of RNA, cDNA Synthesis and Reverse Transcription PCR

Total RNA was extracted from pools of 20 adult *Drosophila* flies using Trizol reagent (MRC, Cincinnati, OH, USA), according to the manufacturer’s instructions. Extracted RNA samples were treated with Turbo DNase (Ambion, Foster City, CA, USA) to remove genomic DNA and the treated RNA was used to generate first-strand cDNA using oligo-dT20 primers with Superscript III reverse transcriptase (Invitrogen, Carlsbad, CA, USA).

Reverse transcription PCR was performed in order to confirm transgenic expression in the progeny. One microliter of cDNA was used in the PCR reaction using specific transgene primers as well as primers for RPL11 (ribosomal protein L11), which served as a reference gene (Table 1). The conditions of the reactions were 95 °C for 5 min followed by 35 cycles of 95 °C for 30 s, 55 °C for 30 s, 72 °C for 30 s, and a final extension for 2 min.

### 2.7. Toxicity Bioassays

We performed an “adult feeding” bioassay as described in [23]. In brief, to investigate the response to acaricides in *Drosophila*, 20 adult flies (10 males and 10 females) aged 2–4 days per replicate were used for the toxicity assay. Flies were collected in plastic vials and the insecticide was provided to them through a Wettex sponge (or cloth). The insecticide was diluted in 5% sucrose. Each dose was tested in 3 replicates and 5% sucrose alone served as the control. Mortality was scored after 24 h. Five to six concentrations that cause 5–95% mortality were used.

A Chi-squared test was used to assess how well the individual LC_50_ values observed in the bioassays agreed with the calculated linear regression lines, and the results were analyzed with PoloPlus (LeOra Software, Berkeley, CA, USA). The resistance ratio (RR) was calculated by comparing the LC_50_ values from each cross vs. the control (*yw* × HR-GAL4). The RRs were considered significant if the 95% fiducial limits (FL) did not include 1 (i.e., if the lower limit was >1) [49].

## 3. Results

### 3.1. Generation of Transgenic Lines Bearing UAS-CYP392A16 and UAS-TuCPR

Several lines containing single insertions of the relevant transgenes were generated by standard P-element transgenesis. Among the different transgenic lines, two UAS-TuCPR lines (line #32, line #92) and two UAS-CYP392A16 lines (line #9, line #71) demonstrating the strong white phenotype (using eye color intensity as a proxy for overall transgene expression level variability due to insertion position effects) were selected for the generation of transgenic flies that express ectopically both CYP392A16 and TuCPR.

A series of genetic crosses (as per the general outline shown in Figure 1) was performed to generate double-responder lines bearing a UAS-TuCPR transgene at chromosome 2, along with a UAS-CYP392A16 transgene at chromosome 3. Thus, four “double-responder” lines were generated, i.e., lines UAS-TuCPR32; UAS-CYP392A16.9, UAS-TuCPR32;UAS-CYP392A16.71, UAS-TuCPR92; UAS-CYP392A16.9, and UAS-TuCPR92;UAS-CYP392A16.71.

In a similar fashion, two “driver-responder” lines were generated bearing the HR-GAl4 driver at chromosome 2 along with the UAS-CYP392A16 transgene at chromosome 3, i.e., lines HR-GAL4;UAS-CYP392A16.9 and HR-GAL4;UAS-CYP392A16.71.

### 3.2. GAL4/UAS Transgenic Co-Expression of CYP392A16 and TuCPR Confers Resistance to Abamectin

**Figure 2:**
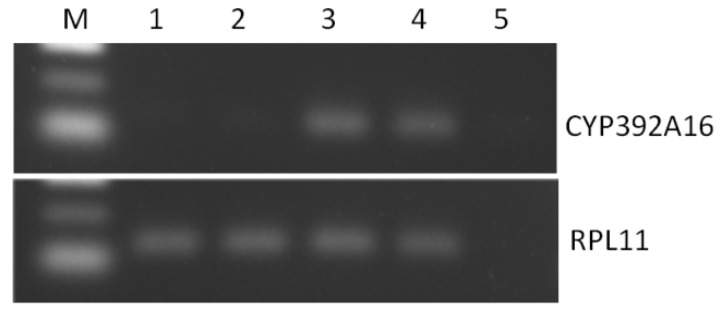
Confirmation of CYP392A16 expression in transgenic *Drosophila* melanogaster by PCR amplification of cDNA. Lanes **1** and **2** represent biological replicates of progeny from the cross *yw* x HR-GAL4 (not expressing CYP392A16) while lanes **3** and **4** are progeny from the cross UAS-TuCPR32; UAS-CYP392A16.71 × HR-GAL4, while lane **5** is a non-template negative control. The top gel represents products amplified with the primers CYP392A16F/R (Table 1), while the bottom gel represents products amplified with primers for the RPL11 housekeeping gene.

Responder (UAS) lines were crossed with a HR-GAL4 driver in order to drive the expression of both *CYP392A16* and *TuCPR* in the midgut, Malpighian tubules, and fat body. Successful expression in the progeny was confirmed by reverse transcription PCR, indicating the presence of the *CYP392A16* transcript in whole adult flies (indicative gels shown in Figure 2).

A series of “feeding” bioassays were conducted using adult progeny (2–4 days post eclosion) from crosses involving different combinations of driver/responder/double-responder lines that correspond to different “dosages”, as shown in Table 2. The survival at different concentrations of abamectin was monitored and the resistance ratio of each line combination versus the *yw* × HR-GAL4 negative control with the same genetic background is shown in Table 2.

The effect of CYP392A16 expression in abamectin-induced mortality was assessed, showing a statistically significant difference (95% fiducial limits not including 1) between certain fly genotypes that overexpress CYP392A16 and the control genotype (Table 2). As shown, CYP392A16 expression is able to confer significant resistance only when TuCPR is co-expressed. Though TuCPR co-expression is not sufficient for significant resistance in every transgene combination, expression of CYP392A16 alone in the absence of TuCPR (i.e., coupling with the endogenous *Drosophila* CPR) does not produce resistant phenotypes at any transgene combination, even with two copies of HR-GAL4 driver and/or UAS-CYP392A16 responder.

## 4. Discussion

Our results indicate that *CYP392A16*, a P450 gene that has been associated with abamectin resistance in *T. urticae* and is capable of metabolizing abamectin in vitro [22], is also capable of conferring resistance in vivo, following expression in detoxification-related tissues in transgenic *Drosophila*. This powerful and versatile system has been used frequently in recent years to test candidate genes for their potential to confer resistance, taking advantage of its unique properties [39,40,41].

In order to systematically assess the role of TuCPR and CYP392A16 in vivo in the absence of confounding resistance mechanisms, we used *D. melanogaster* as a model to induce conditional (GAL4/UAS) expression. We generated a number of strains conditionally expressing CYP392A16 in the presence of endogenous *Drosophila* CPR as a redox partner, or along with TuCPR, and crossed them with HR-GAL4 drivers driving expression in tissues relevant to detoxification, using different driver or responder transgene dosage. We confirmed that the progeny of UAS-CYP392A16 × HR-GAL4 successfully express CYP392A16 and the toxicity bioassays indicated that the transgenic expression of CYP392A16 in *Drosophila* confers abamectin resistance in comparison to control flies with the same genetic background, but only when TuCPR was co-expressed. While TuCPR co-expression does not confer significant resistance in every possible transgene combination, it is possibly critical for the generation of a functional and efficient monooxygenase complex.

Heterologous expression of P450s, both in vitro and in vivo, requires high yields of stable and active P450 enzyme in a functional monooxygenase complex with its redox partner. Several insect P450s have been functionally expressed in vitro in the presence of CPR originating from different insect species/orders [50,51,52,53], demonstrating efficient in vitro metabolism. In principle, CPRs originating from different organisms (mammalian, yeast, or insect) perform the same function and should be interchangeable in heterologous expression systems [34]. Nevertheless, in certain cases, like CYP392A16 and CYP392A11 from *T. urticae*, employment of a “generic” insect CPR, like the relevant enzyme of *Anopheles gambiae* (AgCPR) that has a demonstrated ability to form active complexes with insect P450s [50], was not advantageous, while co-expression of the homologous CPR leads to active P450 enzyme complexes [22,23].

Studies involving in vivo functional validation of insect P450s in *Drosophila melanogaster*, using its endogenous CPR as redox partner, indicate in most cases a resistant phenotype [43,47,51,52,53,54,55,56,57,58] and enable functional validation of candidate P450s. However, the obtained resistance ratios are quite lower than those normally observed in insect pest populations and the absence of the homologous/cognate CPR is a potential system drawback [43].

In the case of *T. urticae*, functional expression of mite P450s in vivo showed a resistant phenotype only in the presence of the homologous TuCPR, both in vitro [22,23] and in vivo [23], and this study. Thus, it is possible that the endogenous *D. melanogaster* CPR might not be able to provide strong resistance phenotypes facilitating efficient validation of mite P450s, presumably due to suboptimal coupling. The potential to form functional and efficient complexes with mite P450s may be compromised given the significant evolutionary distance between insects and mites.

Our findings provide further functional evidence for the role of CYP392A16 in abamectin resistance and show that this approach can be a useful tool for validating candidate spider mite resistance genes, provided that a functional redox partner like TuCPR is provided and the active enzyme complex reconstitution is facilitated. It must be noted, however, that even with this approach, the observed resistance ratios among different strains vs. the control vary from 1.69 to 2.32 at the maximum (Table 2). While such ratios are statistically significant, they only represent a small fraction of observable abamectin resistance in the field [11,17,59,60,61]. This fact either represents an inherent limitation of the *Drosophila* model in order to fully recapitulate the field conditions (also relevant in the assessment of target-site resistance [42]) or reflects the synergistic action of multiple molecular mechanisms in resistant pest populations, perhaps involving target-site abamectin resistance [14], in order to generate the resistant phenotype.

Indeed, research involving investigation of the synergistic interactions of enzyme overexpression and/or target-site mutations within a *Drosophila*-engineered unbiased framework has indicated that the synergistic action of different molecular mechanisms has a multiplicative effect in phenotype manifestation, at least for pyrethroids [44]. This implies that such an experimental system can be readily engineered, for example by stably integrating a TuCPR-expressing transgene together with an attP landing site for ΦC31 integrase or equivalent, which would minimize the “noise” induced by position effects. Further development and optimization of *Drosophila*-based systems for efficient validation of spider mite P450s and assessment of their synergistic action with co-existing resistance mechanisms holds potential for significant insights, towards the elucidation of the complex resistance phenotypes found in pest populations.

## 5. Conclusions

In conclusion, this study has established that *CYP392A16*, a cytochrome P450 from the two-spotted spider mite *T. urticae*, which is capable of metabolizing abamectin in vitro, is also able to confer resistance in vivo as shown by transgenic expression in *Drosophila*. We have also demonstrated that the resistant phenotype is manifested only in the context of TuCPR co-expression, indicating that an evolutionary less divergent partner may be more appropriate for the generation of a functional and efficient monooxygenase complex. Although other resistance mechanisms also have roles in resistance phenotypes found in field populations of *T. urticae*, this information is valuable towards the development of a research framework involving investigation of the synergistic interactions of enzyme overexpression and/or target-site mutations in a *Drosophila*-engineered, unbiased context.

## Figures and Tables

**Table 1:** Primers used in this study.

Primer Name	Sequence (5′–3′)	Product Size (bp)	Reference
CYP392A16_F	AAATACCGAGGTCGGACGTA	117	[20]
CYP392A16_R	AAGCACTTTTTCAATCTGGTCAC		
RPL11_Dm_F	CGATCCCTCCATCGGTATCT	120	[45]
RPL11_Dm_R	AACCACTTCATGGCATCCTC		
pUASTF	TATGTCACACCACAGAAGTAAG	n/a	[47]
pUASTR	CAAGTAAATCAACTGCAACTACTG		

**Table 2:** Abamectin toxicity bioassay responses of transgenic flies expressing CYP392A16 coupled with endogenous *Drosophila* CPR or along TuCPR, with different transgene copy number (dosage) combinations, compared to the control cross *yw* × HR-GAL4 of the same genetic background.

Cross		Transgene Dosage			LC_50_ (95% FL) (mg/L)	Slope	χ^2^ (df)	RR ^1^ (95% FL)
Female	Male	Gal4	CPR	A16				
*yw*	HR-GAL4	1	-	-	45.5 (33.3–56.9)	1.9 ± 0.35	12.1 (16)	
UAS-CYP392A16.71	HR-GAL4	1	-	1	53.4 (38.7–76.2)	1.316 ± 0.25	17.8 (16)	1.17 (0.81–1.71)
UAS-TuCPR92; UAS-CYP392A16.71	HR-GAL4	1	1	1	85.2 (78.6–92.7)	7.1 ± 1.6	10.9 (16)	**1.88** (1.44–2.44)
UAS-TuCPR32; UAS-CYP392A16.71	HR-GAL4	1	1	1	101.8 (88.6–127.6)	3.7 ± 0.62	10.2 (15)	**2.24** (1.65–3.04)
HR-GAL4; UAS-CYP392A16.71	HRGAL4; UAS-CYP392A16.71	2	-	2	45.07 (37.85–51.91)	3.41 ± 0.43	7.98 (10)	0.99 (0.74–1.33)
UAS-TuCPR32; UAS-CYP392A16.71	HR-GAL4; UAS-CYP392A16.71	1	1	2	77.01 (65.99–89.73)	4.37 ± 0.47	14.4 (10)	**1.69** (1.29–2.23)
UAS-CYP392A16.9	HR-GAL4	1	-	1	28.3 (10.3–42)	2.1 ± 0.4	40.3 (15)	0.62 (0.42–0.93)
UAS-TuCPR92; UAS-CYP392A16.9	HR-GAL4	1	1	1	54.4 (40.1–64.3)	4.9 ± 0.8	30.3 (16)	1.19 (0.89–1.59)
UAS-TuCPR32; UAS-CYP392A16.9	HR-GAL4	1	1	1	82.7 (61.7–117.5)	3.1 ± 0.4	49.6 (13)	**1.82** (1.37–2.42)
HR-GAL4; UAS-CYP392A16.9	HR-GAL4; UAS-CYP392A16.9	2	-	2	31.06 (18.82–40.36)	3.2 ± 0.5	14.88 (9)	0.68 (0.79–0.96)
UAS-TuCPR32; UAS-CYP392A16.9	HR-GAL4; UAS-CYP392A16.9	1	1	2	105.29 (79.91–135.06)	4.6 ± 0.6	17.4 (8)	**2.32** (1.74–3.08)

^1^ Statistically significant resistance ratios are shown in bold.
